# The overdiagnosis nightmare: a time for caution

**DOI:** 10.1186/1472-6874-9-34

**Published:** 2009-12-16

**Authors:** Stefano Ciatto

**Affiliations:** 1 Verona Local Health Unit Screening Programme, Marzana Hospital, Piazza R. Lambranzi, 1, 37142 Marzana - Verona, Italy

## Abstract

Overdiagnosis (and overtreatment) of cancers not bound to become symptomatic during lifetime is an unavoidable drawback of mammography screening. The magnitude of overdiagnosis has been estimated to be in the range of 5-10%, and thus acceptable in view of screening benefits as to reduced mortality. In a recent research article in BMC Women's Health, Jørgensen, Zahl and Gøtzsche suggest that overdiagnosis may be as high as 33%, based on their analysis of breast cancer incidence in screened and non-screened areas in Denmark. Here we consider how reliable such analyses can be, why it might have been useful to adjust comparisons between screened and non-screened areas for early detection lead time, and what further evidence might be needed to build on or confirm these results.

## Commentary

In the accompanying article Jørgensen, Zahl and Gøtzsche claim that overdiagnosis generated by screening is as high as 33%, based on an analysis of breast cancer incidence in Danish regions covered and uncovered by population based mammography screening. This is not the first report of a high overdiagnosis level attributed to mammography screening by the authors, who have claimed even higher levels in other countries [1]. Essentially because of overdiagnosis, definitely a negative aspect of screening, the authors suggest that mammography screening might do more harm than good. Such a statement sounds revolutionary in an European scenario where the role of screening efficacy in reducing mortality has long been demonstrated by a number of randomized studies and their meta-analyses. The magnitude of the reported reductions in mortality (about 30-40% in screened vs. non-screened in the 50-69 years age range) has justified a strong recommendation by the European Community [2] that population based screening by biennial mammography should be implemented throughout the Community territory. Such a process has been initiated in all EC countries and full coverage with a homogeneous protocol has already been achieved in many of these countries (e.g. UK, NL, S, FIN).

That overdiagnosis is a necessary and unavoidable drawback of any screening policy for the early detection of cancer, nobody can deny. Of course the magnitude of overdiagnosis depends on several variables, such as indolent, not aggressive cancer prevalence at the screened cancer site, screening test detection lead time, screening aggressiveness, and life expectancy related to screening age.

That overdiagnosis would be a major problem could be easily predicted with prostate cancer screening, as all the favouring conditions for overdiagnosis were present. Autopsy studies showed a prevalence of prostate cancer ranging from 30 to 80% in men dying from other causes [3]. Average detection lead time has been estimated to be in the range of 10-12 years [4]. PSA, the screening test, is positive in 12-15% of healthy screened subjects and prompts random multiple biopsy of the whole prostate [5]. The average screening age is 65, accounting for an average further life expectancy of 15 years (Italy). Overdiagnosis has been estimated to be 50% or higher, depending on screening aggressiveness [4,6]. As any urologist knows, even in absence of an efficient population based screening policy, poorly efficient spontaneous, opportunistic screening caused an unsurprising true epidemic of prostate cancer throughout the western world. In the USA incidence more than doubled and peaked in 1992, with a similar trend observed in Australia and in other western countries. In Florence spontaneous PSA use was not common before 1990 and compliance to PSA driven biopsy was as low as 15-20% [7]. Despite this, the standardized (Europe) incidence rate of prostate cancer (age 55+) in Florence increased from 97.9 in 1985 to 297.9 in 2005 (+204%), with an increasing trend since 1990 [Tuscany Cancer Registry: http://www.ispo.toscana.it/rtrt/statistiche/sede.html, last accessed as of 2009-11-15].

Breast cancer is a different story. Autopsy studies [8] show a much lower prevalence of invasive and *in situ *cancer (1.3% and 8.9%, respectively). Average detection lead time has been estimated to be in the range of 2-3 years [9,10]. The rate of breast biopsies (core-biopsy or surgical) prompted by screening is at most 2-3% [11]. The average screening age is 60, and average further life expectancy exceeds 20 years (Italy). Since screening was introduced no epidemic similar to that seen for prostate cancer has been seen for breast cancer, although a major shift in stage occurred. Overdiagnosis has been estimated to be of much lesser magnitude than suggested by Jørgensen and colleagues, based on data from efficacy trials (Gothenburg and Two Counties = 1% [12]; NBSS I (Canada) = 14% [13]; NBSS II = 11% [13]; Edinburgh = 13% [13]) and from screening services (Florence = 0-13% [14,15]). The limited magnitude of such a negative effect of screening was never considered to outweigh its benefits. In Florence, where population based screening was implemented in 1990, the standardized (Europe) incidence rate of breast cancer (age 50-69) rose from 178.2 in 1985 to 279.0 in 2005 (+56%), with a substantially stable trend [Tuscany Cancer Registry]. A similar trend, with peaks at screening rounds, was observed in most western countries after a national policy was implemented.

The best way to estimate overdiagnosis is to look at cancer incidence before and after screening. According to an ideal model, screening should start and then stop: a peak of incidence due to screen detection will be followed by a drop, possibly below the expected underlying incidence in absence of screening (adjusted by pre-screening trend). Overdiagnosis should be estimated after sufficient time has elapsed since screening stopped, to allow for lead time effect (2-3 years) to subside. When no overdiagnosis is present, excess incidence following screening onset should be fully compensated by the incidence drop following the stopping of screening. The higher the overdiagnosis, the higher the incidence peak at screening onset, the deeper the post-screening drop in incidence. However, this is what occurs in an ideal model, which is likely not the case with the scenario studied by Jørgensen and colleagues. In fact screening did not stop in the screened areas (either the official programme or spontaneous screening), and screening detected (anticipated or overdiagnosed) cancers continued to be added to the observed incidence figures. This has probably also occurred beyond the age of 70: even if screening invitations stop at 69, regular responders until that age are likely to continue their mammography controls. Not adjusting for lead time would lead to overestimating overdiagnosis. It is worth noting that in the study by Jørgensen and colleagues, where a late drop of incidence occurred (for example, as in Funen) suggesting that 70-79 year olds in that area did actually stop having mammography, overdiagnosis estimate dropped to 19%. This may add evidence to the principle that overdiagnosis estimates should be adjusted by lead time. It would be interesting to know what the mammography use was in 70-79 year olds in both Funen and also in Copenhagen where no late drop of incidence was seen, but such data was not available.

Geographic comparisons are tricky. The baseline assumption which makes geographic comparisons reliable is that compared areas are identical as to variables associated to the study outcome, that is the incidence. Again, it is unclear that this is the case with the scenario studied by Jørgensen and colleagues. Apart from the statement by the authors that "Danish population is one of the most homogeneous in the world", reassuring statistical figures (e.g. education, census, parity habits, proportion of urban and rural areas) are not provided, and having the "second largest city" and "rural areas" does not necessarily equate to non-screened and screened areas. Baseline pre-screening (1971-1990) incidence is similar, being only 8% higher in screened as compared to non-screened areas (screening age core group). After 1991 incidence increases substantially in non-screened areas (+44% as compared to 1971-1990). We don't know how much of this is due to opportunistic screening (no data on mammography use are provided) or to causes other than opportunistic screening (e.g. hormone replacement therapy (HRT), changes in lifestyle or reproductive habits, usually occurring one or two decades before). Due to the masking effect of screening, we ignore what would have been the spontaneous trend in incidence in screened areas, and we can not be sure that it would be the same as in non-screened areas. Indeed, had causes other than screening (e.g. HRT use, changes in lifestyle or reproductive habits) been more prevalent in the screened areas, this would cause a higher, screening unrelated underlying incidence and would also lead to overdiagnosis overestimation.

In summary, the evidence provided by Jørgensen and colleagues is not yet fully convincing. It does not adjust for lead time, which tends to overestimate overdiagnosis. It is also based on the assumption that considered screened and non-screened areas are comparable as to underlying incidence, whereas no detailed supporting evidence of their comparability (e.g. risk factors) is provided to support their case. The authors' challenge to the European Community recommendation of implementing population-based mammography screening, and their message that screening might do "more harm than good" could be considered to be based on some unproven assumptions. The "good" is well established by randomized trials and population screening outcomes. Some of the "harm" is unavoidable with screening and overdiagnosis is part of that, but the message that the magnitude of such "harm" may counterbalance the "good" is not yet confirmed and is countered by several other studies on overdiagnosis which give estimates between 0 and 13% [11-14].

## Competing interests

The author declares that they have no competing interests.

## Pre-publication history

The pre-publication history for this paper can be accessed here:

http://www.biomedcentral.com/1472-6874/9/34/prepub

## References

[B1] Jørgensen KJ Gøtzsche PC Overdiagnosis in publicly organized mammography screening programmes: Systematic review of incidence trends Br Med J 2009 339 b2587 10.1136/bmj.b2587 PMC2714679 19589821

[B2] Perry NM Broeders M de Wolf C Törnberg S Holland R von Karsa L European guidelines for quality assurance in breast cancer screening and diagnosis 2006 Fourth Luxembourg: European Commission 10.1093/annonc/mdm481 18024988

[B3] Holund B Latent prostatic cancer in a consecutive autopsy series Scand J Urol Nephrol 1980 14 29 43 6154966 10.3109/00365598009181186

[B4] Draisma G Boer R Otto SJ Cruijsen IW van der Damhuis RA Schröder FH Lead times and overdetection due to prostate-specific antigen screening: estimates from the European Randomized Study of Screening for Prostate Cancer J Natl Cancer Inst 2003 95 868 78 12813170 10.1093/jnci/95.12.868

[B5] Schröder FH Hugosson J Roobol MJ Tammela TL Ciatto S Nelen V Screening and prostate-cancer mortality in a randomized European study N Engl J Med 2009 360 1320 8 10.1056/NEJMoa0810084 19297566

[B6] Zappa M Ciatto S Bonardi R Mazzotta A Overdiagnosis of prostate carcinoma by screening: an estimate based on the results of the Florence Screening Pilot Study Ann Oncol 1998 9 1297 300 10.1023/A:1008492013196 9932159

[B7] Ciatto S Houssami N Martinelli F Giusti F Zappa M PSA use and incidence of prostate biopsy in the Tuscany Region: is opportunistic screening discounting biopsy in subjects with PSA elevation? Tumori 2008 94 518 22 18822688 10.1177/030089160809400413

[B8] Welch HG Black WC Using autopsy series to estimate the disease "reservoir" for ductal carcinoma in situ of the breast: how much more breast cancer can we find? Ann Intern Med 1997 127 1023 8 9412284 10.7326/0003-4819-127-11-199712010-00014

[B9] Jonsson H Johansson R Lenner P Increased incidence of invasive breast cancer after the introduction of service screening with mammography in Swedennt J Cancer 2005 117 I842 7 10.1002/ijc.21228 15957172

[B10] Duffy SW Lynge E Jonsson H Ayyaz S Olsen AH Complexities in estimation of overdiagnosis in breast cancer screening Br J Cancer 2008 99 1176 8 10.1038/sj.bjc.6604638 18766185 PMC2567065

[B11] Zappa M Spagnolo G Ciatto S Giorgi D Paci E Rosselli Del Turco M Measurement of the costs in two mammographic screening programmes in the province of Florence, Italy J Med Screen 1995 2 191 4 8719147 10.1177/096914139500200404

[B12] Duffy SW Agbaje O Tabar L Vitak B Bjurstam N Björneld L Overdiagnosis and overtreatment of breast cancer: estimates of overdiagnosis from two trials of mammographic screening for breast cancer Breast Cancer Res 2005 7 258 65 1-5 gotehnburg two county 10.1186/bcr1354 16457701 PMC1410738

[B13] Moss S Overdiagnosis and overtreatment of breast cancer: overdiagnosis in randomised controlled trials of breast cancer screening Breast Cancer Res 2005 7 230 4 10.1186/bcr1314 16168145 PMC1242166

[B14] Puliti D Zappa M Miccinesi G Falini P Crocetti E Paci E An estimate of overdiagnosis 15 years after the start of mammographic screening in Florence Eur J Cancer 2009 in press 19879130 10.1016/j.ejca.2009.06.014

[B15] Paci E Warwick J Falini P Duffy SW Overdiagnosis in screening: is the increase in breast cancer incidence rates a cause for concern? J Med Scree 2004 11 23 7 5 10.1258/096914104772950718 15006110

